# Small spaces, big impacts: contributions of micro-environmental variation to population persistence under climate change

**DOI:** 10.1093/aobpla/plaa005

**Published:** 2020-02-18

**Authors:** Derek A Denney, M Inam Jameel, Jordan B Bemmels, Mia E Rochford, Jill T Anderson

**Affiliations:** 1 Department of Plant Biology, University of Georgia, Athens, GA, USA; 2 Department of Genetics, University of Georgia, Athens, GA, USA; 3 Department of Biological Sciences, University of Toronto Scarborough, Toronto, ON, Canada

**Keywords:** climate change, ecophysiology, genetic variation, local adaptation, microenvrionment, microhabitat, paleorefugia, plasticity, refugia

## Abstract

Individuals within natural populations can experience very different abiotic and biotic conditions across small spatial scales owing to microtopography and other micro-environmental gradients. Ecological and evolutionary studies often ignore the effects of micro-environment on plant population and community dynamics. Here, we explore the extent to which fine-grained variation in abiotic and biotic conditions contributes to within-population variation in trait expression and genetic diversity in natural plant populations. Furthermore, we consider whether benign microhabitats could buffer local populations of some plant species from abiotic stresses imposed by rapid anthropogenic climate change. If microrefugia sustain local populations and communities in the short term, other eco-evolutionary processes, such as gene flow and adaptation, could enhance population stability in the longer term. We caution, however, that local populations may still decline in size as they contract into rare microhabitats and microrefugia. We encourage future research that explicitly examines the role of the micro-environment in maintaining genetic variation within local populations, favouring the evolution of phenotypic plasticity at local scales and enhancing population persistence under global change.

## Teaser

The environment can vary at small spatial scales, such that neighbouring plants can grow under different resource levels and experience different degrees of competition. This fine-grained biotic and abiotic variation in the environment could favour the maintenance of genetic diversity within populations. Climate change is rapidly altering complex suites of environments to which natural populations have adapted. Here, we discuss the influence of the micro-environment on physiology, adaptation and plasticity in the context of novel and rapidly changing environments. We consider the role of micro-environments as paleorefugia during previous climatic shifts, and the potential of existing micro-environmental variation to serve as refugia under contemporary climate change.

## Introduction

Environmental conditions vary across the distribution of a species, both at the macroscale, such as across broad latitudinal or elevational gradients, and the microscale, such as microtopography within a local site. Macro- and micro-environmental variation exposes natural populations to mosaics of resource availability, differing abiotic conditions, and a range of biotic interactions. In this review, we explore the extent to which local micro-environmental variation influences phenotypic plasticity, patterns of genetic variation and population persistence, especially in the context of global change. Fine-grained heterogeneity in topography, nutrient levels, water available and other environmental conditions creates microhabitats. Soil microhabitats can differ substantially in microbiotic diversity within small regions (Smith *et al.* 2011). Small-scale topographic features such as slopes and washes of ephemeral desert river beds can dramatically influence plant physiology (Ehleringer and Cooper 1988). In tropical forests, rooting phenotype differs substantially in species inhabiting canopy gaps versus understory sites (Paz 2003). Micro-environmental variation can influence tree and shrub recruitment and plant distribution patterns (Anderson 2009; Anderson *et al.* 2009). Furthermore, animal activities often create micro-environments that influence plant community composition, species diversity and phenotypic variation; for example, ant or kangaroo rat mounds (Guo 1998) or beaver dams (Wright *et al.* 2002) alter abiotic conditions such as soil moisture content, light availability or nitrogen content in the soil.

**Figure 1.**
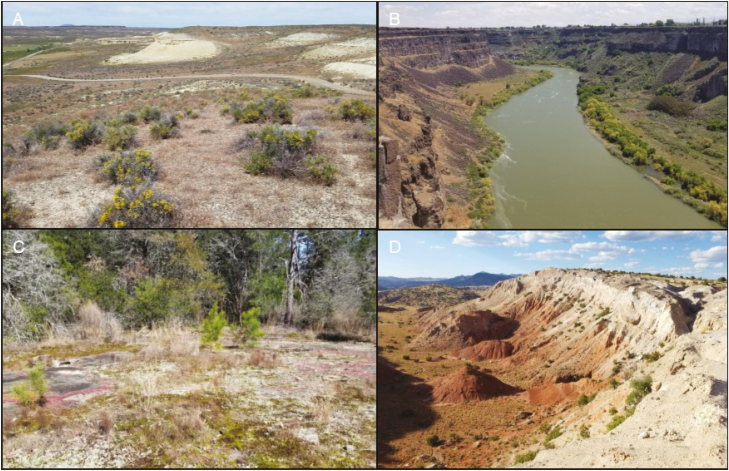
Examples of micro-environmental variation. (A) Soil ionic properties change drastically, as shown by zeolite outcrops in south-eastern Oregon, USA. (B) Slope and aspect influence light intensity along the Snake River, Idaho, USA. (C) Surface temperatures and soil depth are affected by the granite outcrops of Rock and Shoals, Georgia, USA. (D) The Tierra Amarilla Anticline of northern New Mexico, USA, is composed of sandstone and gypsum soils, which affect water availability to plants.

We define a plant’s micro-environment as the small-scale abiotic and biotic conditions the individual experiences, which may differ from average conditions experienced by other individuals within the same population. Some locales may contain discrete microhabitats that can easily be distinguished from the surrounding environment, such as treefall gaps within a forest (Paz 2003) or elevated microsites within wetlands (Anderson 2009; Anderson *et al.* 2009). In other habitats, the micro-environment varies continuously across microtopographic and other gradients (Box 1; Fig. 1). Crucially, micro-environmental variation has the potential to buffer natural populations from the immediate effects of global climate change, thereby enabling populations to persist locally (De Frenne *et al.* 2013; McLaughlin *et al.* 2017).

Box 1: Quantifying micro-environmental variation. (A) Discrete microhabitats have historically been identified by observing variation in species distributions, such as saguaro cacti seedlings growing under shade provided from nurse plants (Turner *et al.* 1966) or species growing on different soil types such as serpentine soil communities (Kruckeberg 1951) and gypsum outcrops (Meyer 1986). Researchers have quantified micro-environmental variation by measuring photosynthetically active radiation (PAR), pH, soil surface temperatures and soil nitrogen content in small regions of a landscape near plants of interest (Franco and Nobel 1989). Others have included soil-level details about microhabitat including microbe presence, isotopic composition of the soil (Smith *et al.* 2011) or soil texture (Misiewicz and Fine 2014). To characterize microhabitats regionally through time, one can deploy data loggers to track air and soil temperature, humidity, light levels, edaphic factors (e.g. pH, temperature, moisture) over multiple spatial and temporal scales (Fawcett *et al.* 2019). When combined with other thermal quantification techniques, such as infrared imaging, data loggers in the soil can be used to recover surface and air temperature variation across slopes of mountainsides (Scherrer and Koerner 2010). Recent technological advances have led to new techniques in remote sensing and identification of environmental variation. Counter-intuitively, remote sensing tools can provide fine-resolution information to help identify microrefugia as well as assess the quality and stability of the habitat when used with similarly fine-scale environmental data (Andrew and Warrener 2017). Landsat data have been used to monitor vegetation dynamics and may be used to detect and predict microrefugia (Andrew and Warrener 2017). Aerial Light Detection and Ranging (LiDAR) can detect microhabitat variation across a landscape and develop raster layers of soil composition for use in geographic information systems (Allie *et al.* 2015) while terrestrial LiDAR has been employed to map understory habitats to identify microhabitat variation within forest ecosystems (Tymen *et al.* 2017). New advancements in terrestrial laser scanning (TLS) can construct fine-scale 3D maps of object surfaces to the millimetre level (Rehush *et al.* 2018). Nevertheless, heterogeneity makes it difficult to use landscape features to predict microrefugia, so other fine-scaled observational tools are still necessary (Andrew and Warrener 2017). The tools required to delineate discrete microhabitats are becoming readily available and the computer algorithms designed to process the information are emerging quickly (Graham *et al.* 2019). These technological advances are beyond the purview of our paper; however, they are reviewed extensively elsewhere (Zellweger *et al.* 2019). (B) This hypothetical raster of a landscape illustrates the details that emerge from small-scale environmental data. As environmental data become more readily available, we can better inform our models with greater accuracy (Connor *et al.* 2019).

**Figure:**
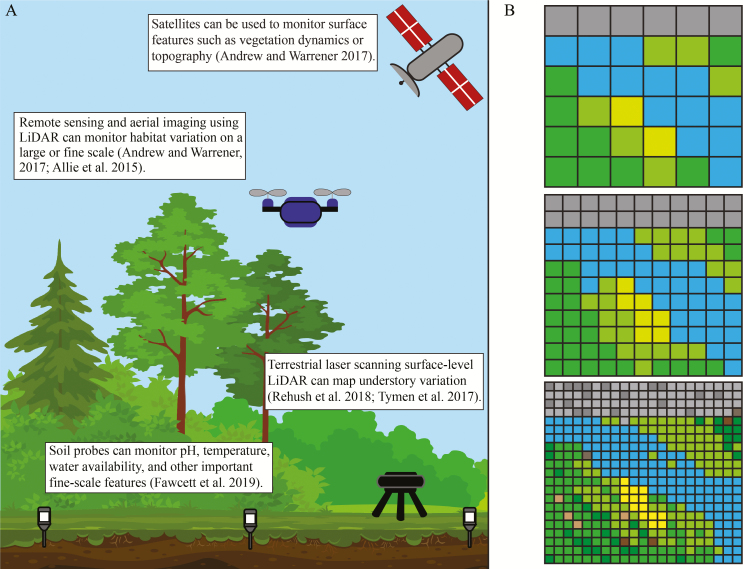

In this review, we explore how micro-environmental variation influences the factors that contribute to phenotypic variation within natural populations, and the implications of this phenotypic variation for populations’ responses to climate change. Phenotypic variation arises through genetic variation, environmental variation (plasticity) and genotype-by-environment interactions. Climate change has influenced all of these components of phenotypic variation (e.g. Franks *et al.* 2007; Nicotra *et al.* 2010; Wilczek *et al.* 2014; Anderson and Wadgymar 2019). Industrialization and other human activities have disrupted the global climate (IPCC 2013), thereby decoupling previously correlated conditions (like temperature and photoperiod) that drive life history evolution (e.g. Wadgymar *et al.* 2018b). Climate is a potent agent of selection on natural populations (e.g. Siepielski *et al.* 2017), and climate change is likely exerting novel patterns of selection by altering the abiotic and biotic environment (e.g. Van der Putten *et al.* 2010; Becklin *et al.* 2016). Some plant traits, like physiological parameters, are highly responsive to environmental conditions and will rapidly adjust to environmental change. In other cases, micro-environmental variation within contemporary landscapes may have already favoured adaptive genetic variation that could enable population persistence under climate change. To that end, this review examines how the micro-environment could influence how plant populations respond to novel stresses imposed by climate change. Specifically, we investigate plant physiology and phenotypic plasticity, along with genetic variation within populations through the lens of micro-environmental variation. We end by describing how micro-environmental variation could create microrefugia, which have conserved populations through geological episodes of climate change and which could be critical assets for conservation under rapid anthropogenic climate change.

### Physiology, climate change and micro-environment

**Figure 2.**
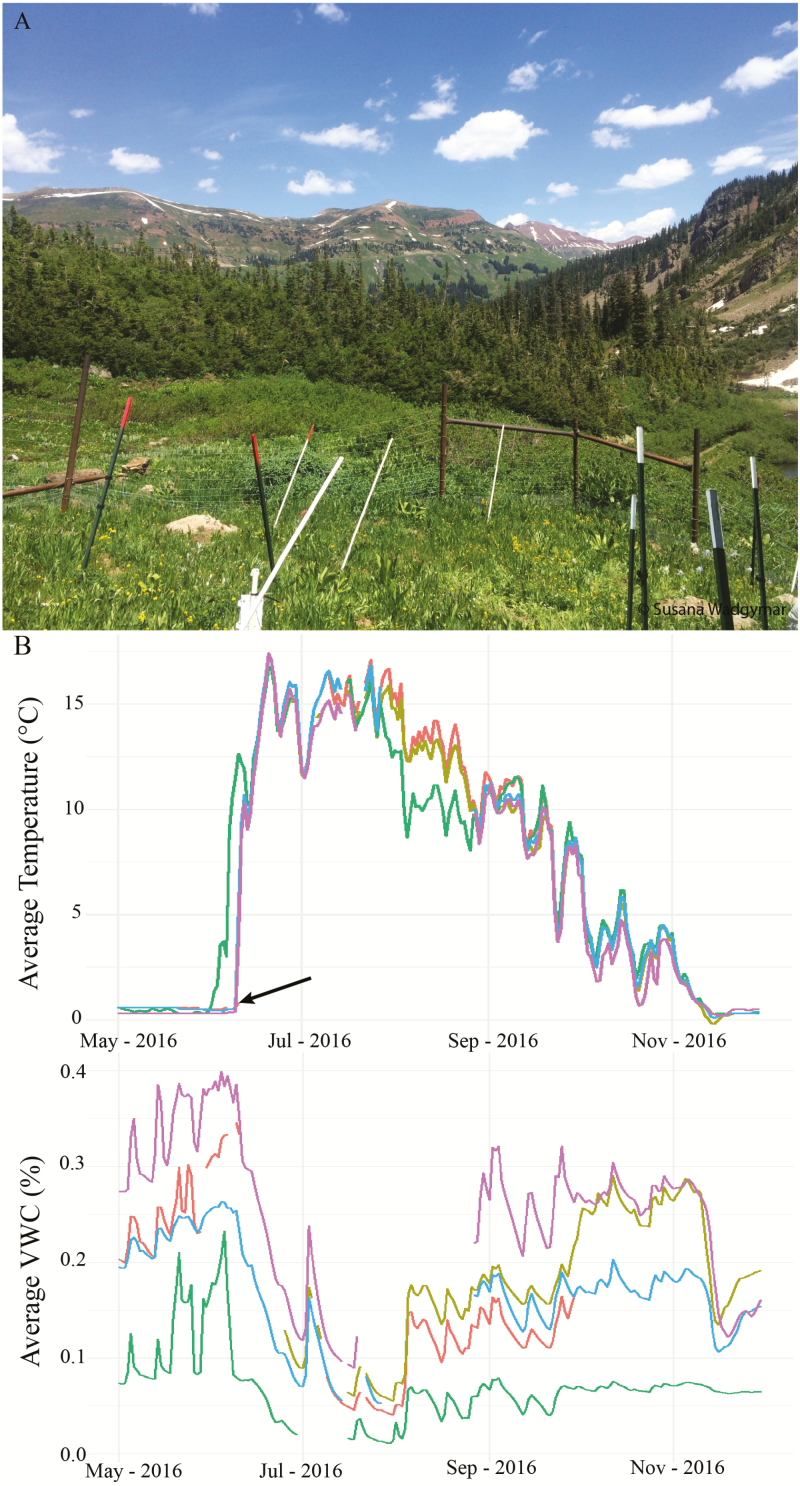
Micro-environmental variation can affect soil temperatures and water availability across small geographic areas. Here, we present soil temperature and volumetric water content as logged by five soil probes within 2 m of one another buried at a depth of 10 cm within a common garden site at the Rocky Mountain Biological Laboratory (A; elevation 3340 m). (B) Each color represents one probe. The data loggers record measurements at 15-min intervals; this figure displays data from the 2016 growing season. The arrow indicates the final day of snowmelt in the garden. Missing data can occur from gophers tampering with the soil probes. Although the probes are within close proximity to one another, there is a significant difference in mean annual soil temperature and volumetric water content across the garden site. By measuring small-scale differences in habitats, we can understand more about the environmental heterogeneity of regions and how these factors will affect plant populations. Climatic data displayed can be found in Supporting Information—Table S1.

Anthropogenic climate change and other human activities are strongly influencing abiotic conditions as well as natural communities (Mitchell and Whitney 2018). Climate change has increased global temperatures, altered precipitation patterns and augmented the frequency of extreme weather events, with the extent of these changes differing across regions (Knapp *et al.* 2015; Borghi *et al.* 2019). Climate change and other anthropogenic forces act across broad scales, yet their effects on plants will depend on how they influence the local environments that individuals experience (Siepielski *et al.* 2017). The abiotic and biotic stressors plants encounter in their microhabitats affect physiological, phenological and morphological traits (Pereira 2016; Biswas *et al.* 2019). Water availability can vary substantially at fine spatial scales (Fig. 2), such as across an ephemeral riverbed along a well-drained slope (Free *et al.* 2013) or along elevational gradients (Kooyers *et al.* 2019). North- and south-facing slopes of mountainsides can experience stark differences in surface and air temperature (as much as 6 °C in the Swiss Alps) and soil temperature (3–5 °C in the Kluane region of Canada) (Scherrer and Koerner 2010; Dearborn and Danby 2017). Microhabitats can vary in light availability (Cervantes *et al.* 2005) and edaphic factors like metal concentrations (Ginocchio *et al.* 2004) and soil composition (Boege and Dirzo 2004). Thus, one plant may experience stress due to limited water availability, excessive waterlogging, thermal extremes or soil salinity, while a neighbour occupying a different micro-environment may experience more benign conditions. Understanding how microhabitats influence physiology will be essential to predicting the ecological and evolutionary responses to global change, and evaluating the potential for microhabitats to protect populations from some of the negative effects of this change.

Water availability plays a large role in the expression of physiological traits such as photosynthesis, water-use efficiency, stomatal conductance and stomatal density (Farooq *et al.* 2012). Water stress can reduce stomatal conductance and inhibit the biochemical pathways of photosynthesis (Tezara *et al.* 1999; Misson *et al.* 2010; Mathobo *et al.* 2017), but the extent to which a plant responds to drought and the type of response are a product of its genotype and its environment (Chaves *et al.* 2003). Even within biomes, precipitation patterns can determine plant species distributions (Engelbrecht *et al.* 2007), but it is more challenging to predict future precipitation under climate change than temperature patterns (Reside *et al.* 2019). Some models project that precipitation will increase at high latitudes, and that extreme conditions such as flooding or drought will become more pronounced in the tropics (Dore 2005; Schwartz *et al.* 2019). However, models differ in their projections about shifts in the intensity, frequency and duration of precipitation events (Trenberth 2011), making it challenging to predict how climate change will influence broad-scale precipitation patterns (Matte *et al.* 2019).

As models improve, predictions about regional-scale precipitation changes may become more accurate, yet understanding how these regional-scale changes influence local plant populations will be incomplete unless studies account for local micro-environments. Much research has examined plant responses to drought stress from the cellular to the organismal and even to the ecosystem level (Flexas *et al.* 2012; Pessarakli 2016), and experiments often attempt to scale from a few individuals measured under controlled conditions to the overall effect of drought on a region (Walker *et al.* 2018; Schwartz *et al.* 2019). Nonetheless, site-specific heterogeneity influences a plant’s response to drought. For example, *Populus* species (Salicaceae) grown in a semi-arid environment showed substantial variation in stomatal size and density, attributed to small-scale climatic conditions in localized micro-environments (Pearce *et al.* 2006). Future experiments examining drought should incorporate micro-environmental variation if we are to assess the role of microhabitats in buffering against climate change. Recent technological advances have allowed us to consider this approach on a larger scale. For example, LiDAR analyses of a tropical forest’s response to drought in Puerto Rico indicated that micro-environmental conditions such as solar radiation and moisture-bearing trade winds outweighed the effects of topography (slope, aspect and elevation) in forest recovery from drought, contrary to predictions (Schwartz *et al.* 2019).

In addition to affecting water dynamics, climate change is projected to continue to increase temperatures globally and cause more frequent heatwaves and temperature extremes (Hatfield and Prueger 2015). Thermal stress will disrupt the biochemical pathways related to photosynthesis (Kumarathunge *et al.* 2019), and can result in leaf senescence, decreased yield (in agricultural systems) and fitness (in natural populations), and inhibited root or shoot growth (Wahid *et al.* 2007). However, the degree of thermal stress depends on the species, phenological stage and the plant’s ability to process temperature fluctuations on a daily basis (Źróbek-Sokolnik 2012). For example, seedlings from four species of Chihuahuan Cactaceae and three species from Asparagaceae showed species-specific effects on chlorophyll fluorescence and relative growth rate when grown in full sunlight (a heat-stress treatment) versus under the shade of mesquite nurse plants (*Prosopis laevigata*, Fabaceae) (Pérez-Sánchez *et al.* 2015). Attempts to generalize the relationship between temperature and tree mortality on a regional scale in the Sierra Nevada Mountains (Paz-Kagan *et al.* 2017) as well as characterize plant traits on a global scale have repeatedly indicated physiological mechanisms for coping with temperature stress are species- and habitat-specific (Liu *et al.* 2017). Temperature varies substantially with micro-environmental conditions such as wind, light intensity, water supply and daylength (Kozlowski *et al.* 1991). These factors are often considered environmental noise (Bertolli and Souza 2013). However, modelling environmental variability has increased crop breeder’s ability to predict and select for phenotypes across heterogeneous landscapes (Jarquín *et al.* 2014; Monteverde *et al.* 2019; Rincent *et al.* 2019). Additionally, when ecological niche models account for fine-scale environmental data, they can generate more robust predictions of species distribution patterns in contemporary (Pradervand *et al.* 2013) and future climates (Lenoir *et al.* 2017). By quantifying micro-environmental variation such as temperature, we can develop more effective models of individual physiology and ecosystem processes under climate change (Pincebourde *et al.* 2016; Tymen *et al.* 2017).

The effects of the abiotic stresses discussed above have mostly been evaluated in isolation; however, elevated [CO_2_], increased temperature and disrupted precipitation patterns interact to alter plant physiology (Xu *et al.* 2013; Ficklin and Novick 2017; Chiang *et al.* 2018). Open-air and growth chamber manipulations have revealed that increasing CO_2_ can induce higher photosynthetic rates (Farquhar 1997; Ainsworth and Rogers 2007; Bagley *et al.* 2015; Dusenge *et al.* 2019), and reduced stomatal conductance (Kirschbaum and McMillan 2018), which could increase plant growth rate in an environment with more carbon dioxide (Xu *et al.* 2016). Warming temperatures can also enhance photosynthetic capacity of leaves (Linkosalo *et al.* 2016); however, an increase in carbon production may not necessarily augment biomass (Morrison and Lawlor 1999). Conversely, increasing temperatures are projected to intensify drought stress (Ficklin and Novick 2017), and elevated [CO_2_] combined with hotter temperatures may exacerbate heat stress (Warren *et al.* 2011), potentially counteracting any fitness gains from higher photosynthetic rates. Plant responses to simultaneous changes in temperature, aridity and CO_2_ concentration may differ substantially from predictions generated by studying each factor in isolation (Zandalinas *et al.* 2018). Plant physiological and demographic responses to changing abiotic conditions depend on habitat heterogeneity and biotic interactions across microhabitats (Tomiolo *et al.* 2015; Kleynhans *et al.* 2016). As such, experiments considering the effects of climate change should account for the fine-grained interactive responses between abiotic and biotic conditions (Price *et al.* 2013). Multifactorial experiments provide robust tests of how climate change factors interact in contemporary landscapes (Eller *et al.* 2011). These interactive forces will likely vary across the landscape.

Micro-environmental variation could potentially protect local populations from the adverse physiological effects of climate change (Silva *et al.* 2013). Uriarte and colleagues (2018) monitored seedling growth and survival within a tropical forest in Puerto Rico over a 9-year period, finding that moist microhabitats buffered individuals against the negative effects of fluctuating rainfall and solar radiation. Likewise, in the semi-arid rangelands of Mongolia, fine-grained variation in the environment maintains functional trait variation within local communities, which could potentially enhance population persistence under novel precipitation patterns and increased temperatures (Lang *et al.* 2019). Thus, fine-scale environmental heterogeneity may mitigate the effects of climate change if climatically benign microsites exist locally (McLaughlin *et al.* 2017).

#### Future directions

 Micro-environmental variation affects plants from the cellular to the organismal level by influencing temperature, nutrient availability and water availability. Modelling fine-grained environmental variation could lead to a more profound understanding of the dynamics that shape physiological plasticity and influence the genetic structure of populations across landscapes (Thuiller *et al.* 2008). Challenges arise when models use large grid sizes that overshadow fine-scale heterogeneity (Thuiller *et al.* 2008). Nevertheless, recent approaches have sought to address these difficulties with a combination of fine-grained environmental data and more readily available coarse-grained data sets (Mertes and Jetz 2018). Additionally, researchers are developing advanced geographic information system data sets that incorporate fine-scale environmental variation (Graham *et al.* 2019). We encourage future multifactorial experiments that evaluate the consequences of changing micro-environmental conditions on plant physiology. Such studies can test the hypothesis that fitness gains from elevated [CO_2_] are counterbalanced by fitness losses from increasing temperatures, novel precipitation regimes and micro-environmental stresses. Additionally, multifactorial manipulations may reveal ways in which micro-environments could ameliorate the effects of climate change.

The increasing availability and cost-effectiveness of applying large-scale genomic, transcriptomic and epigenomic analyses to non-model organisms (Singhal 2013; Ellegren 2014; Richards *et al.* 2017) will also improve our understanding of physiological responses to microhabitat variation in natural plant populations. Differences in gene expression may underlie plant responses to variation in moisture regime, temperature, soil types and other environmental gradients (e.g. Sork 2017; Gould *et al.* 2018; Tripathi *et al.* 2019). Likewise, epigenetic variation may differentiate plant populations inhabiting different microhabitats (Richards *et al.* 2012; Herrera and Bazaga 2016), suggesting that epigenetic mechanisms could also contribute to physiological responses to climate change. In particular, future genomic, transcriptomic and epigenomic studies could identify the molecular bases for physiological responses of natural populations to projected future climatic conditions, and whether different microsites may potentially strengthen or dampen these responses.

In addition to affecting the abiotic conditions that directly influence plant physiology, climate change will also alter biotic interactions among species, including competition, pollination, herbivory and host–pathogen dynamics (Box 2). We summarize predictions regarding many of these biotic interactions in Box 2, yet an exhaustive consideration of the impacts of climate change on community dynamics is beyond the scope of this review. Nonetheless, microsites have the ability to alter biotic interactions over small spatial scales (Fransen *et al.* 2001; Collins and Foré 2009; Soliveres *et al.* 2015). Studies aiming to predict how climate change will affect community dynamics ought to include consideration of microhabitat variation and its potential to strength, weaken or fundamentally alter the impacts of climate change on biotic interactions.

Box 2: Overview of environmental shifts impacting plant communities as a result of climate change. As the effects of climate change progress, plant communities will encounter novel biotic and abiotic. Below is a synopsis of the most pertinent challenges they will face and a non-exhaustive list of references.

**Table:** 

Effect	Prediction	References
**Abiotic**		
[CO_2_]	Increased: CO_2_ concentrations are increasing by ~20 ppm per decade due to anthropogenic forces.	IPCC (2013), Bereiter *et al.* (2015), Lüthi *et al.* (2008)
Temperature	Increased: Globally, temperatures will increase, and more frequent heat waves and temperature extremes will become the norm. Night-time temperatures are projected to increase more than daytime temperatures. Soil temperatures are warming faster than air temperature.	Hatfield and Prueger (2015), Karl *et al.* (1991), Zhang *et al.* (2016)
Precipitation	Altered: High latitudes will experience an increase in precipitation. All areas are projected to see an increase in extreme conditions such as flooding or drought associated with altered precipitation patterns.	Dore (2005), Schwartz *et al.* (2019), Trenberth (2011)
Soil	Altered: Cation exchange capacity will be altered, and soils will become more acidic. C and N cycling in the soil will be affected by increased temperatures.	Allen *et al.* (2011), Rengel (2011)
		
**Biotic**		
Resource competition	Altered: Certain species will gain a competitive advantage under climate change, whereas others will be at a disadvantage, which could shift community dynamics.	Urban *et al.* (2012), Blois *et al.* (2013), Gilman *et al.* (2010), Alexander *et al.* (2015)
Plant community composition	Altered: Woody shrubs will encroach upon grasslands and move poleward. Novel plant communities may arise due to differing migratory potential.	Gilman *et al.* (2010), Bond and Midgley (2012), Blois *et al.* (2013), Pearson *et al.* (2013), Urban *et al.* (2012)
Pollinator–plant interactions	Disrupted: Flowering phenology and pollinator activity respond to different environment cues, and potentially become unsynchronized. Flowering may decrease due to insufficient vernalizing temperatures.	Hegland *et al.* (2009), Byers (2017), Inouye (2019), Byers and Chang (2017)
Herbivory	Increased: Insect herbivores could adapt quickly to effects of climate change, consume more plant material as C:N ratios in leaves increase and expand ranges into herbivore-naïve plant communities. Increased opportunities for mammalian and insect herbivory across longer growing seasons.	Liu *et al.* (2011), Rasmann *et al.* (2014), Robinson *et al.* (2012), Becklin *et al.* (2016)
Below-ground interactions	Altered: Soil microbial community composition can be affected by drought conditions and carbon levels determined through leaf litter. Precipitation patterns can also determine top-soil microbial diversity.	Kaisermann *et al.* (2017), Pugnaire *et al.* (2019), Sheik *et al.* (2011), Eisenhauer *et al.* (2012)

### Genetic variation within populations

As climate change intensifies, the persistence of local populations depends on the extent of heritable genetic variation in traits subject to novel selection imposed by climate change (Hughes *et al.* 2008; Bellard *et al.* 2012; Salmela 2014; Wilczek *et al.* 2014; Ravenscroft *et al.* 2015; Becklin *et al.* 2016; Sheth *et al.* 2018). Many plant species have adapted to local abiotic and biotic conditions across habitat types or broad spatial scales where gene flow is limited (Leimu and Fischer 2008; Lowry *et al.* 2008; Hereford 2009; Wadgymar *et al.* 2018a). In addition, micro-environmental variation can exert divergent selection across small spatial scales even *within* natural populations, resulting in microgeographic adaptation (Schmitt and Gamble 1990; Argyres and Schmitt 1991; Lechowicz and Bell 1991; Antonovics 2006; Dittmar and Schemske 2017). Could microhabitats maintain sufficient genetic variation in functional traits for populations to adapt to climate change (Bridle and Vines 2007; Richardson *et al.* 2014; Becklin *et al.* 2016; Gossmann *et al.* 2019; Razgour *et al.* 2019)?

If microhabitat heterogeneity causes the magnitude and direction of selection to vary extensively, pockets of genetic variation could be maintained within local populations, as long as localized selection is strong enough to overcome gene flow (Steiner and Berrang 1990; Argyres and Schmitt 1991; Richardson *et al.* 2014). For example, *Impatiens* species (Balsaminaceae) in the forest understory demonstrate adaptive differentiation between populations located at very fine spatial scales (10–50 m) (Schemske 1984; Schmitt and Gamble 1990; Argyres and Schmitt 1991). Indeed, local populations have differentiated genetically in response to micro-environmental variation in edaphic conditions (Lechowicz and Bell 1991). Additionally, temporal variation can maintain genetic diversity within populations (Salmela *et al.* 2013; Salmela 2014; Salmela *et al.* 2016). For example, in *Pinus sylvestris* (Pinaceae), populations encountering variable yearly growing seasons have greater genetic variation in reproductive phenology than populations in more stable conditions (Salmela *et al.* 2013; Salmela 2014).

Discrete microhabitats that differ substantially in abiotic or biotic conditions from the surrounding environment can contain individuals with alleles or traits that are not common in the rest of the population (Richardson *et al.* 2014; Prentice *et al.* 2015). For example, populations of the outcrossing grass *Anthoxanthum odoratum* (Poaceae) occur in both heavy metal contaminated soils and pasture habitats, separated by a distance of <20 m (Antonovics 1972, 1976, 2006). Common garden experiments have revealed that populations from these two soil types in close proximity are genetically differentiated in metal tolerance (Antonovics 1968, 2006; McNeilly and Antonovics 1968), and plants from contaminated soils flower earlier than plants in nearby, relatively benign pasture soil (Antonovics 1968, 2006; McNeilly and Antonovics 1968; Antonovics and Bradshaw 1970). Similarly, in reciprocal transplant experiments, populations of *Leptosipon parvifloris* (Polemoniaceae) from serpentine soils flowered earlier than populations from sandstone soils in both habitat types, indicative of genetic differentiation across short spatial scales, as these habitats can be separated by a distance of <10 m (Kay *et al.* 2011; Dittmar and Schemske 2017). Reproductive phenology also differs between *Ricotia lunaria* (Brassicaceae) populations inhabiting contrasting microsites within the Evolution Canyon model system in Lower Nahal Oren, Mount Carmel, Israel (Kossover *et al.* 2009; Nevo 2012; Qian *et al.* 2018). *Ricotia lunaria* inhabits both the warm, dry, open savannah-like south-facing slope and the lush, green, shaded temperate north-facing slope, which are separated by only 100 m at the base (Nevo 2012). In common garden environments, populations from the south-facing slope flowered earlier than those on the north-facing slope, and showed upregulation of drought-response genes (Kossover *et al.* 2009; Nevo 2012; Qian *et al.* 2018). Thus, fine-grained spatial heterogeneity in the landscape can selectively favour adaptive genetic variation within populations.

The genetic difference in flowering of metal-tolerant *A. odoratum*, serpentine-adapted *L. parviflorvis* and drought tolerant *R. lunaria* may become more relevant under climate change. The early onset of flowering often enables plants to escape from drought stress, which is expected to increase in severity as a result of climate change (Franks 2011; Dai 2013; Weis *et al.* 2014). Furthermore, in some species, climate change favours advanced flowering (Franks *et al.* 2007; Munguía-Rosas *et al.* 2011; Anderson *et al.* 2012; Bemmels and Anderson 2019). As a result, populations of *A. odoratum*, *L. parviflorvis*, *R. lunaria* and other species that are adapted to rare edaphic conditions could contribute to the continued local persistence of their species in the context of climate change (Richardson *et al.* 2014).

Genetically diverse populations may have increased adaptive potential under environmental change, which is increasingly important as human activities continue to fragment natural habitats and climate change alters abiotic and biotic conditions. For example, *Festuca ovina* (Poaceae) is a widespread perennial grass that grows across the various microhabitats on the island of Öland (Sweden) (Prentice *et al.* 1995, 2015). In addition to one native copy of the gene coding for the enzyme PGIC (*PgiC1*), some *F. ovina* populations also have an additional expressed transgene copy (*PgiC2(f)*) horizontally derived from the genus *Poa* (Poaceae), which may help them adapt to micro-environmental variation in pH and soil moisture (Vallenback *et al.* 2008, 2010a, b; Prentice *et al.* 2015). PGIC plays a key role in glucose metabolism, and polymorphisms in PGIC are associated with variation in environmental factors such as temperature and salinity (Riddoch 1993; Prentice *et al.* 2015). In this system, the populations harbouring the transgene along with the native copy could have enhanced capacity to adapt to an increasingly fragmented landscape under climate change (Prentice *et al.* 2015).

To generate robust predictions of evolutionary responses to environmental change, studies can examine how populations respond genetically to climatic manipulations through time (e.g. Davies and Snaydon 1976; Panetta *et al.* 2018), resurrect historical lineages for comparison with current populations (e.g. Franks *et al.* 2008; Gómez *et al.* 2018) or expose contemporary populations to historical, current and predicted future conditions (e.g. Anderson and Wadgymar 2019). Longitudinal studies are uniquely situated to evaluate adaptive potential in future climates if they monitor populations in treatments relevant to climate change. As part of the long-standing Park Grass Experiment, *A. odoratum* (Poaceae) was maintained in a mosaic formation of plots differing in nutrient levels for ~60 years (Davies and Snaydon 1976; Silvertown *et al.* 2006). The plots were spaced closely such that gene flow was expected to occur; nevertheless, reciprocal transplant experiments found that populations of *A. odoratum* were adapted to their local nutrient conditions, indicating that adaptation in this species can be rapidly achieved via standing genetic variation (Davies and Snaydon 1976; Silvertown *et al.* 2006). Thus, fragmented populations of *A. odoratum* may be able to adapt to rapid deterioration of environmental conditions due to climate change. Resurrection studies that compare populations before and after episodes of climate change (e.g. Franks *et al.* 2018; Hamann *et al.* 2018) can also accomplish similar objectives of assessing adaptive potential of natural populations. For example, Franks and colleagues (Franks *et al.* 2007; Hamann *et al.* 2018) documented rapid adaptation of drought escape in the mustard *Brassica rapa* (Brassicaceae) by comparing lineages collected before and after severe droughts in California. Despite rapid evolution of flowering time and other traits associated with drought escape, contemporary generations express lower fitness than historical generations, suggesting that climate change may quickly outpace adaptive evolution, even for short-lived species (Hamann *et al.* 2018). Taken together, these approaches provide powerful tests of the adaptive potential of natural populations under climate change.

#### Future directions

**Figure 3.**
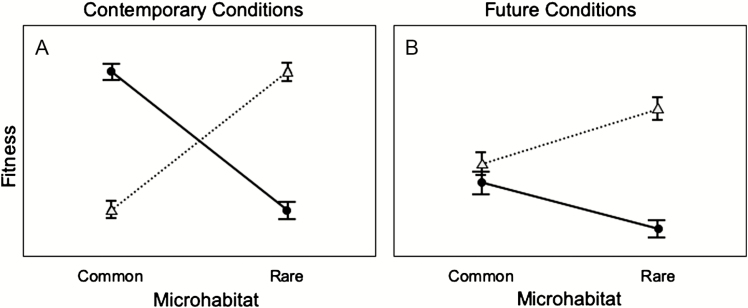
Rare genotypes can display a fitness advantage over common genotypes in future conditions. Panels show fitness reaction norms of common and rare genotypes in contemporary and future conditions. Solid lines with circles refer to fitness values of common genotypes and dashed lines with triangles represent the fitness values of rare genotypes. Common and rare genotypes display higher fitness in their relative microhabitats under contemporary conditions (panel A). In future conditions, rare genotypes can have a fitness advantage through ‘pre-adaptation’ to stressful conditions by harbouring unique adaptive alleles (panel B). Rare and common genotypes can also decline in fitness overall in future conditions due to factors such as elevated temperatures, increased drought, increased herbivory and habitat fragmentation.

 The genetic variation present within locally adapted populations may enable populations to withstand environmental changes associated with climate change (Razgour *et al.* 2019). In contrast, if limited gene flow restricts migration, locally adapted populations could be increasingly vulnerable to novel climatic conditions (Anderson and Wadgymar 2019). Studies that account for fine-scale micro-environmental variation could identify populations potentially harbouring sufficient genetic variation to persist under climate change. Indeed, Sexton and colleagues (2013) found that environmental factors had a greater influence on genetic structure within populations than geographic distance across 70 published studies. Landscape genetic analyses using individual-based sampling that incorporate micro-environmental variation can provide insight into the ways in which heterogeneous landscapes affect genetic variation (Wang and Bradburd 2014). Local populations may already include individuals that are adapted to conditions consistent with climatic projections for the area. These pre-adapted lineages could contribute disproportionately to adaptive responses to climate change (Fig. 3).

The rate of adaptation in a population is proportional to its additive genetic variance for fitness (Fisher 1930; Shaw and Etterson 2012); thus, high genetic variance in fitness could aid populations in adapting to climate change. However, empirical studies assessing the additive genetic variance for fitness and evolutionary potential of natural populations are rare (Hendry *et al.* 2018; but see Sheth *et al.* 2018; Bemmels and Anderson 2019). Estimates of the additive genetic variance for fitness can be statistically and methodologically difficult to obtain (Hendry *et al.* 2018), but studies aiming to predict evolutionary dynamics under climate change should estimate these parameters for populations exposed to projected future climates whenever possible. Researchers should also quantify whether differences in additive genetic variance for fitness exist between populations from heterogeneous and homogenous environments to gain further insight into whether historical exposure to micro-environmental variation has influenced populations’ future adaptive potential.

 In many cases, estimates of additive genetic variance for fitness may be unattainable, but researchers can examine genetic variation in functional traits relevant to climate change across a variety of microhabitats. For example, flowering phenology is highly responsive to climatic variation, and climate change may impose selection favouring earlier flowering (Sherry *et al.* 2007; Cleland *et al.* 2012; Wadgymar *et al.* 2018b; Bemmels and Anderson 2019). Other functional phenotypes that could generate novel insights about climate change responses include foliar traits related to adaptation to increased temperatures and drought stress (specific leaf area, leaf water content, stomata size and density, water-use efficiency), and traits related to herbivore defence and resistance, as herbivory may increase with warmer temperatures (Anderson and Gezon 2015; Arnold *et al.* 2019). Additionally, drought stress can favour the evolution of smaller flowers (e.g. Galen 2000) to reduce water loss. As pollinators typically prefer larger flowers (e.g. Sandring and Agren 2009; Parachnowitsch and Kessler 2010; Krizek and Anderson 2013; Lavi and Sapir 2015), drought-mediated selection for smaller flowers could fundamentally alter plant–pollinator interactions in outcrossing species. Genotypes from distinct microhabitats displaying trait differentiation can be exposed to contemporary and projected future conditions in common gardens and other experiments under field conditions (e.g. Bemmels and Anderson 2019) to assess the degree to which a trait is genetically or environmentally determined, to evaluate genetic variation in complex traits, and to test the adaptive nature of these traits. Additionally, global change is not expected to uniformly influence the microclimate of different soil types (Hamidov *et al.* 2018). The selective pressures existing across distinct soil types can vary, possibly resulting in key adaptations to future environmental conditions.

Genomic scans have successfully identified signatures of selection in many populations (Jump *et al.* 2006; Manel *et al.* 2010; Yeaman *et al.* 2016; Leempoel *et al.* 2018). Intriguingly, these studies have occasionally detected signatures of local adaptation at very fine (i.e. microsite-level) spatial scales, including in *Salix herbacea* (Cortés 2017) and in animals (e.g. a salt-marsh fish, Wagner *et al.* 2017). Researchers can make additional inferences about adaptive population divergence by analysing fitness data. For example, in the annual forb, *Arabidopsis thaliana* (Brassicaceae), Price *et al.* (2018) identified specific genes responsible for genetic trade-offs and described signatures of divergent evolutionary trajectories by combining information on QTLs (quantitative trait loci) previously mapped for fitness with population genomic methods (Price *et al.* 2018). Reciprocal transplant experiments for studying local adaptation can be conducted in conjunction with genomic scans that employ fine-scale population sampling, an assessment of microhabitat environmental heterogeneity, estimates of gene flow among microhabitats and knowledge of variation in functional traits. This research programme would provide a comprehensive picture of a population’s genetic potential to adapt to climate change conditions and the extent to which microsites facilitate the maintenance of adaptive genetic variation within populations.

### Phenotypic plasticity

In addition to adapting to novel conditions via genetic changes in trait means across generations, populations respond to climate change through phenotypic plasticity (Nicotra *et al.* 2010). Many plant species have evolved plasticity in one or more traits, such that families with high levels of plasticity readily shift their phenotypes in response to the environment they encounter (Bradshaw 1965; Via and Lande 1985; Schlichting 1986; Sultan 1987; Mooney and Agrawal 2008). Adaptive plasticity is a strategy that maximizes fitness across habitat types and environmental gradients. Fine-grained spatial and temporal heterogeneity in the environment can favour the evolution of phenotypic plasticity if individuals experience multiple abiotic or biotic conditions across their lifespans or if their offspring disperse into distinct microhabitats (Moran 1992; Stratton and Bennington 1998; Alpert and Simms 2002). Micro-environmental variation increases the degree of selection for phenotypic plasticity in a population, and therefore the ability of plasticity to contribute to population persistence under climate change. One example where microhabitats have been linked to the evolution of plasticity is in the annual plant *Erodium cicutarium* (Geraniaceae), which inhabits both serpentine and non-serpentine soils in California (USA) (Baythavong and Stanton 2010; Baythavong 2011). The serpentine microhabitats of *E. cicutarium* consist of heterogeneous micro-environments located well within typical dispersal distances of one another, whereas the non-serpentine soils are more consistent in edaphic conditions and feature fewer distinct microsites (Baythavong and Stanton 2010; Baythavong 2011). Divergent selection favours increased plasticity in the serpentine soils and reduced plasticity in the non-serpentine, producing patterns of genetic differentiation in this system (Baythavong and Stanton 2010; Baythavong 2011). Micro-environmental variation can thus favour the evolution of adaptive plasticity, which could allow populations to respond rapidly to future climates if those axes of environmental variation currently exist within microhabitats.

Phenotypic plasticity itself is a trait under genetic control and can evolve in response to selection (Schlichting 1986; Sultan 1987; Mooney and Agrawal 2008; Baythavong and Stanton 2010). Several studies have quantified heritability in plasticity by evaluating genotype-by-environment interactions in key functional traits (Scheiner and Lyman 1989; Scheiner 2002; Nussey *et al.* 2005; Relyea 2005; Visser 2008; Anderson and Gezon 2015). For example, the subalpine forb *Boechera stricta* (Brassicaceae) maintains significant heritability in plasticity of phenological and morphological traits (Anderson and Gezon 2015). Through calculating the heritability of traits and plasticity, it is possible to make predictions about the extent to which populations will respond to novel selection imposed by climate change, and whether plasticity has the potential to evolve.

Adaptive plasticity could buffer populations from the effects of climate change in the short term (Chevin and Lande 2010; Chevin *et al.* 2010; Nicotra *et al.* 2010). Populations have already shifted their geographic distributions in response to climate change (e.g. Grabherr *et al.* 1994; Kelly and Goulden 2008; Lenoir *et al.* 2008; Chen *et al.* 2011; Fadrique *et al.* 2018; Steinbauer *et al.* 2018). However, climate change could rapidly outpace the adaptive and migratory potential of most species (Billington and Pelham 1991; Hoffmann *et al.* 2003; Loarie *et al.* 2009; Bellard *et al.* 2012; Wilczek *et al.* 2014; Gossmann *et al.* 2019). Plasticity could prevent short-term population declines if individuals can shift their trait values in response to novel climates until migration or adaptation can occur (Nicotra *et al.* 2010; Fox *et al.* 2019). Microhabitat variation thus has the potential to contribute to persistence in rapidly changing conditions through favouring the evolution of plasticity.

Populations that have historically been exposed to high microhabitat heterogeneity may have evolved higher phenotypic plasticity and thus be better equipped to respond to changing climates than populations from stable, homogenous environments. However, not all examples of plasticity are adaptive (Langerhans and DeWitt 2002; Price *et al.* 2003; Ghalambor *et al.* 2007; Chevin and Lande 2015). When plasticity causes traits to shift in a direction antagonistic to the direction of selection, plasticity can be maladaptive. Maladaptive plasticity can arise as a response to environmental stress (Ghalambor *et al.* 2007; Chevin and Lande 2015). For example, stem elongation to escape from the shade of competitors is usually an adaptive phenotype (Dudley and Schmitt 1996; Schmitt *et al.* 1999; Weinig and Delph 2001). However, when the competitors are too tall to overcome, stem elongation can result in a fitness cost for the organism, resulting in maladaptive plasticity (Weinig 2000; Steinger *et al.* 2003). Whether plasticity in response to climate change will be adaptive or maladaptive may depend on the specific trait and environmental context.

Adaptive plasticity evolves in fine-grained heterogenous environments with high levels of gene flow, which can hinder local adaptation (Schlichting 1986; Sultan 1987; Mooney and Agrawal 2008; Baythavong and Stanton 2010). For example, in the Australian woody shrub *Dodonaea viscosa* (Sapindaceae), populations separated by short geographic distances and connected by extensive gene flow experience extensive variation in temperature, aridity and precipitation (Baruch *et al.* 2018). Substantial variation in functional traits among populations in close proximity results from phenotypic plasticity rather than local adaptation in this species, allowing *D. viscosa* to have a higher potential to acclimate to climate change (Baruch *et al.* 2018). Similarly, in the salt marsh plant *Borrichia frutescens* (Asteraceae) on Sapelo Island in Georgia, USA, populations located only 20–50 m apart are likely connected via pollen flow and can differ dramatically in phenotype (e.g. height) depending on salt concentration (Richards *et al.* 2010). In *B. frutescens*, trait variation across salt gradients arises from phenotypic plasticity rather than from genetic adaptation (Richards *et al.* 2010). High levels of plasticity in *D. viscosa* and *B. frutescens* could indicate that they can respond to rapid environmental fluctuations as a result of climate change within a single generation. The existence of gene flow over heterogeneous microhabitats is a hallmark for the evolution of adaptive plasticity, though quite often, a mix of genetic adaptation and plasticity occurs in nature (Wadgymar *et al.* 2017a). In systems where gene flow between populations inhabiting different microsites is low, genetic adaptation may be more likely to evolve than phenotypic plasticity, and the response to climate change may be delayed.

Thus far, we have focused on within generation plasticity. However, trans-generational plasticity can also contribute to trait expression when the parental environment strongly predicts the environment that the offspring will experience; in those cases, parents can provision their progeny with extra-genetic information that can enhance offspring fitness in parental environment. Thus, trans-generational plasticity could accelerate phenotypic responses to environmental conditions (Wolf and Wade 2009; Holeski *et al.* 2012; Baker *et al.* 2019). Trans-generational plasticity is expected to be favoured under stable environments but has the potential to be maladaptive if progeny encounter conditions that differ from those experienced by their parents (Galloway 2004; Visser 2008; Wadgymar *et al.* 2018b). The maternal environment influences offspring responses to shade and herbivory (Galloway 2004; Bell and Galloway 2007; Galloway and Etterson 2007; Colicchio *et al.* 2015; Colicchio 2017) and even simulated climate change (Wadgymar *et al.* 2018b). Epigenetic variation may be one mechanism by which plant phenotypic plasticity can rapidly evolve (Zhang *et al.* 2013). More multigenerational experiments are needed to further assess the origins and populational-level effects of trans-generational plasticity (Donelson *et al.* 2018), especially in the context of climate change.

#### Future directions

As a result of climate change, plant populations face rapid shifts in abiotic and biotic stress (Box 2), which may outpace adaptive evolution and migration for many species (Loarie *et al.* 2009). Plasticity could enable local populations to express appropriate trait values rapidly, thereby mitigating the immediate effects of climate change (Nicotra *et al.* 2010). Theory suggests adaptive plasticity evolves in populations that experience fine-scaled temporal and spatial heterogeneity. Future studies should determine whether populations can manifest optimal phenotypes under simulations of projected climates, within a single generation and in a trans-generational context. Transcriptomic and epigenomic studies will be crucial in gaining a mechanistic understanding of how individual plants may plastically respond to changing climates, and whether these plastic responses may be passed to subsequent generations and promote rapid adaptation (Zhang *et al.* 2013).

### Microhabitats as microrefugia: a paleobotanical perspective

Microhabitats influence plant physiology, potentially buffer against environmental change, promote the maintenance of adaptive genetic variation and contribute to the evolution of phenotypic plasticity. However, we do not know whether the small spatial scale of these effects is relevant to predicting long-term responses to global climate change. Do microhabitats facilitate species persistence over centuries to millennia, or are they ephemeral reservoirs for populations doomed to extinction? Microrefugia are microhabitats that allow populations of formerly widespread species to persist locally through periods of inhospitable conditions until a return to favourable regional climates (Rull 2009; Hannah *et al.* 2014). Because microrefugia distinguish local from regional environments and may potentially encompass an entire local population, the spatial extent of microrefugia is likely to be somewhat larger than that of the fine-scale, within-population microhabitat variation that has been the primary focus of our review. However, our discussion of microrefugia continues our consideration of the effects of small-scale environmental variation, in which heterogeneous landscapes create isolated habitats of limited spatial extent that differ markedly from their surroundings.

Empirical paleobotanical and phylogeographic studies have revealed that microrefugia sometimes facilitated survival through major, long-lasting climate shifts such as Pleistocene glacial cycles (Mee and Moore 2014). Pleistocene glaciation induced major changes in plant distributions globally, including latitudinal and elevational shifts, and range contractions and expansions (Bush and Colinvaux 1990; Hewitt 1999, 2000; van der Hammen and Hooghiemstra 2000; Davis and Shaw 2001; Carnaval *et al.* 2009; Dupont 2011; Qiu *et al.* 2011). Traditional paradigms for understanding the distributions of warm-adapted species during glacial periods have emphasized the importance of large refugia where regional-scale climates were suitable for species persistence (Hewitt 1999, 2000; Harrison *et al.* 2001; Soltis *et al.* 2006; Carnaval *et al.* 2009). In temperate forest plants, recent evidence has increasingly challenged this viewpoint and highlighted evidence for long-term persistence of populations in small, scattered microrefugia within areas where regional climates were generally unable to support persistence (Stewart and Lister 2001; Willis and van Andel 2004; McLachlan *et al.* 2005; Provan and Bennett 2008; Rull 2009; Bemmels *et al.* 2019). The concept of microrefugia has also been applied to understanding Pleistocene distributions of animals (Schmitt and Varga 2012), as well as plants from alpine (Patsiou *et al.* 2014), boreal (Väliranta *et al.* 2011) and tropical biomes (Leal 2001; Collins *et al.* 2013).

The precise ecological and edaphic characteristics of microrefugia have rarely been identified (Rull 2009; but see Delcourt *et al.* 1980; Delcourt and Delcourt 1984). However, for a site to serve as a microrefugium, local environmental conditions must be decoupled from regional climates (Dobrowski 2011; Hylander *et al.* 2015; McLaughlin *et al.* 2017). Decoupled environmental conditions are particularly likely to arise in topographically complex environments, where slope, aspect and landscape position strongly affect local climates (Dobrowski 2011; Hylander *et al.* 2015). Hydrologic microrefugia may also exist where local sites are more mesic than surrounding areas (McLaughlin *et al.* 2017). Beyond facilitating population persistence, microrefugia may also have evolutionary consequences (Mosblech *et al.* 2011; Mee and Moore 2014). Small populations are subject to strong genetic drift, suggesting that populations in microrefugia could exhibit reduced genetic diversity and increased inbreeding, and be strongly genetically differentiated from one another and from larger populations in areas of stable regional climates (Mosblech *et al.* 2011; Mee and Moore 2014). Range expansion from microrefugia following a return to favourable climates may therefore have important consequences for genetic diversity in recolonized areas. Regions recolonized by populations that expanded from one or a small number of microrefugia might be expected to exhibit low genetic diversity. Alternatively, simultaneous recolonization from numerous genetically differentiated microrefugia could result in higher overall genetic diversity than would be expected given recolonization from a single, large refugium (Mosblech *et al.* 2011).

Microrefugia are also likely to differ in selection regimes relative to other areas where populations persist, suggesting that survival in microrefugia may drive the evolution of local adaptation (Mee and Moore 2014). Local adaptation is widespread in contemporary plants (Leimu and Fischer 2008) and was likely also prevalent in ancient populations (Davis and Shaw 2001), including microrefugia (Mee and Moore 2014). However, low genetic diversity, high genetic drift and inbreeding depression associated with small populations may reduce the ability of microrefugial populations to adapt to local environments (Leimu and Fischer 2008). In contrast, small population sizes could favour the evolution of specific traits, such as self-fertilization to avoid inbreeding depression (Mee and Moore 2014). Collectively, the independent evolution of populations inhabiting different microrefugia whether due to neutral genetic phenomenon or as a result of selection, and subsequent differential survival and extinction of these populations, may not only allow species to persist through periods of climate change, but may also lead to evolutionary change in the species as a whole (Stewart 2009).

#### Future directions

A general consensus on the relevance of microrefugia to species persistence through extended periods of climate change remains elusive (Hewitt 1999; Stewart and Lister 2001; Tzedakis *et al.* 2013; Rull 2014) for at least two major reasons. Firstly, microrefugia are difficult to detect using traditional paleobotanical and phylogeographic tools (Tzedakis *et al.* 2013), and their existence is often inferred but rarely explicitly documented (Rull 2009). Multiple lines of evidence including fossil evidence, genetic studies and species distribution models have widely been used to infer the presence of refugia, but each approach has a variety of limitations and potential biases; the strengths and weakness of these approaches have been reviewed elsewhere (Tzedakis *et al.* 2013; Gavin *et al.* 2014). Secondly, even in exceptional cases where macrofossils provide physical evidence of localized species presence in a putative microrefugium (e.g. Delcourt *et al.* 1980; de Lafontaine *et al.* 2014), macrofossils do not necessarily represent populations that persisted through an entire period of climate change and left descendants in modern populations. Instead, these populations might eventually have gone extinct (Stewart and Lister 2001; Hannah *et al.* 2014) or otherwise failed to contribute to range recolonization after a return to favourable climates (Mandák *et al.* 2016).

Future research into microrefugia would benefit from combining these multiple lines of evidence (e.g. fossil evidence, genetic studies and species distribution models) to strengthen confidence in the identification of microrefugia (de Lafontaine *et al.* 2014; Gavin *et al.* 2014). In addition, explicitly testing whether small, isolated populations left descendants in modern populations would help clarify that these populations indeed contributed to long-term species persistence, rather than serving as evolutionary dead-ends (Hannah *et al.* 2014). Genomic data may be combined with recent advances in statistical phylogeography (e.g. Carnaval *et al.* 2009; He *et al.* 2013, 2017) to test these hypotheses and to reveal novel and detailed geographic and ecological insights into the roles of microrefugia. For example, modelling methodologies that incorporate genetic, climatological, spatial and ecological information, and that can be statistically evaluated using Approximate Bayesian Computation (Beaumont *et al.* 2002), can provide a robust hypothesis-testing framework. These types of models have been used to identify the latitude and longitude of refugia from which eastern North American hickories recolonized formerly glaciated regions, revealing that one species expanded from northern microrefugia while another species did not (Bemmels *et al.* 2019); to determine that differences in microhabitat affinity drove different responses to glaciation in two co-distributed Rocky Mountain sedges (Massatti and Knowles 2016); and to demonstrate that postglacial range shifts that tracked summer drought regimes at a regional scale better explain genetic structure in a Californian oak than shifts constrained by microhabitat availability (Bemmels *et al.* 2016). As modelling techniques improve, statistical phylogeographic approaches will no doubt continue to provide quantitative support for or against the importance of microrefugia, especially in cases where qualitative interpretation of traditional data sets has been controversial (Tzedakis *et al.* 2013).

Growing evidence that Pleistocene microrefugia likely existed for some, but not all, plant species also highlights the species-specific nature of responses to climate change (Stewart 2009; Stewart *et al.* 2010; Papadopoulou and Knowles 2016). Why some species persisted in microrefugia but others did not remains poorly understood and is an area in need of further study. Theoretical arguments suggest that both the climatic conditions necessary to generate microrefugia and the ability to persist in them could depend strongly on species’ individual ecological niches. In particular, microrefugia are likely to arise only when species’ ranges are limited by climatic factors that are not strongly correlated with other aspects of regional climate (Hylander *et al.* 2015). Traits such as high stress tolerance, long lifespan, asexual reproduction or selfing mating systems, and low genetic load could increase the probability that small plant populations will persist in these environments (Hampe and Jump 2011; Mosblech *et al.* 2011). Empirical evidence has suggested that for temperate woody plants, traits that may have facilitated survival in microrefugia include small seeds, wind dispersal, vegetative reproduction and generalist habitat affinities (Bhagwat and Willis 2008). In addition, microrefugia are more frequently proposed to have existed for mesic and generalist temperate trees than for dry-adapted species (Bemmels *et al.* 2019), but patterns are too preliminary to draw firm conclusions. Finally, the ability to persist in microrefugia may also depend on biotic interactions with other community members, such as competitors, pollinators and pathogens. Climate change may influence the potential geographic distributions of each of these community members differently (Stewart *et al.* 2010; Hylander *et al.* 2015), leading to non-analogue communities and novel biotic interactions (Jackson *et al.* 2000). For example, lowered CO_2_ concentrations during glacial periods could have led to the increased competitive advantage of conifers over broadleaf deciduous trees on a wider variety of microsites, due to the greater carbon-use efficiency of conifers (Jackson *et al.* 2000). However, the potential role of biotic interactions in mediating survival in microrefugia has as yet been underexplored.

### The role of microrefugia in plant conservation

Given that microrefugia have helped facilitate persistence of some plant species in response to historical climate change, could they also play a role in plant conservation in the face of ongoing global change? Benign habitats located within or near the current distribution of a species could provide refuge for local populations in the face of climate change. These refugia could enhance population persistence for populations that lack the genetic variation needed to adapt to novel climates quickly or that have limited migratory potential (Olson *et al.* 2012). Refugia can occur within short distances of current distributions and therefore be accessible to species with spatially restricted seed and pollen movement (Hylander *et al.* 2015). In addition, some refugia will occur in areas that form part of current distributions and will consist of relictual populations (Rull 2009). Because climate change may rapidly alter conditions across broad geographic areas, many of the most readily accessible future habitats within or near current distributions may only exist at small local sites where conditions are decoupled from regional patterns (Hylander *et al.* 2015), highlighting the potential importance of microrefugia.

Conservation planning that incorporates a potential role for microrefugia should be informed by data on functional trait variation within these microsites. Within plant species, functional traits that influence fitness often vary across environmental gradients; this intraspecific trait variation arises through a combination of long-term adaptation to local environments, phenotypic plasticity, genetic drift and other demographic processes (Ackerly *et al.* 2002; Kooyers *et al.* 2015; Wadgymar *et al.* 2017a, b). Plant phenotypes can also vary across micro-environmental gradients within local sites (Blonder *et al.* 2018). For example, in an alpine plant community in the Colorado Rocky Mountains, moist fine-textured soils with high phosphorus and nitrogen levels were associated with long root systems and rapid growth rates at the micro-environmental scale (Blonder *et al.* 2018). Species’ distributions within micro-environments were not only influenced by abiotic conditions, but also by competition with other species in the community (Blonder *et al.* 2018), which can vary in magnitude due to nutrient patchiness in heterogeneous soils (Fransen *et al.* 2001; Bliss *et al.* 2002; Garcia-Palacios *et al.* 2012). Combining abiotic data that captures micro-environmental variation in a region with demographic and trait data (e.g. Oldfather and Ackerly 2019) from observational and experimental studies will improve predictive models of community structure and dynamics, as the micro-environment can determine species distributions (Blonder *et al.* 2018).

In addition to refugia’s role as a climatic haven, these areas might host unique habitat types that support rare species with specific habitat requirements (Wintle *et al.* 2019). For example, tall and short grass prairies in the mid-Western United States were heavily converted to agriculture in the 18th century because of their rich soils (Camill *et al.* 2004). As a result, the native prairies of Minnesota have become rare and occupy <1 % of their original range (Camill *et al.* 2004). Throughout the region, these grasslands only exist now as small and isolated patches, yet prairie specialists require these rare patches for persistence (Camill *et al.* 2004). Rare habitat types are especially susceptible to fragmentation and degradation, as they are uncommon in the landscape. Because these habitats are also likely to contain rare species, these fragments are of high conservation priority and are crucial to the species’ future success (Wintle *et al.* 2019). Small, isolated populations typically have restricted genetic variation, and communities in fragmented landscapes may have high prevalence of invasive species (Wintle *et al.* 2019). Given that >50 % of the world’s terrestrial habitats now exist in a state of intermediate to very high human modification (Kennedy 2018), conservation biology will increasingly require managing populations confined to small habitat patches such as these, highlighting the need for research into the viability of populations in microrefugia and other small populations.

Pervasive anthropogenic habitat fragmentation alters microclimatic conditions within a landscape, thereby increasing the difficulty of identifying potential refugia (Latimer and Zuckerberg 2017). Given an increased impact of edge effects, isolated forest fragments experienced greater temperature fluctuations than large continuous forests. Additionally, current microrefugia are threatened by land-use change (Davies *et al.* 2017). Areas that have been identified as a current or potential refugium are seldom given protection, legal or otherwise. Because microrefugia are still not regarded as holding much conservation value, they face greater risk of disturbance. Restoring degraded sites so that they may serve as refugia is a viable option for plant conservation (Braidwood *et al.* 2018). Threatened plants may be transplanted to sites within or at the edge of the species’ range to ensure their survival during periods of climate change that may cause local extinction (Braidwood *et al.* 2018).

Current methods show promise for successfully identifying stable refugia—areas that are habitable in the long term and are not subject to the environmental fluctuations that surrounding areas may experience (Tang *et al.* 2018). Some areas with a high level of endemism may be climatically stable future refugia (Harrison and Noss 2017). Centres of endemism typically occur in regions that are not subject to large fluctuations in climate, like coastal zones, because these conditions enable the persistence of rare taxa (Harrison and Noss 2017). Mountainous regions typically have high topographical and climatic diversity; this heterogeneity not only fosters biodiversity, but it also could create climate refugia (Harrison and Noss 2017). Models for predicting species’ risk of extinction that combine current and past species distributions, climate, topography and edaphic data are more accurate than models that only take climatic factors and species distribution into account (Niskanen *et al.* 2016). By accounting for micro-environmental variation, models can generate more robust predictions about extinction under climate change (Niskanen *et al.* 2016). It is possible to construct reliable models for predicting the locations of climate refugia given sufficient and appropriately small-scale and species-specific biotic and abiotic data for the local environment (Niskanen *et al.* 2016). For example, Tang and colleagues (2018) combined current and historical species abundances and distributions with climatic data form relictual populations to identify refugia and determine the relative stability of refugia in the face of climate change. Ecological niche models showed that mild winter temperatures generate long-term stable refugia in East Asia (Tang *et al.* 2018). Monitoring endemic species richness across the landscape will also help conservationists prioritize refugia (Tang *et al.* 2018).

#### Future directions

 Conceptually, microrefugia could play a critical role in plant responses to anthropogenic climatic changes by enabling populations to endure without the need to migrate long distances to track rapidly changing climates (Neilson *et al.* 2005; Rull 2009; Feurdean *et al.* 2013). Conservationists still debate which criteria to use when identifying refugia, making it challenging to implement policy or management practices (Ashcroft 2010). For example, it is uncertain whether refuges will be of higher conservation value if they best reflect the historical climate of a region or the fundamental niche of the species inhabiting the region (Ashcroft 2010).

**Figure 4.**
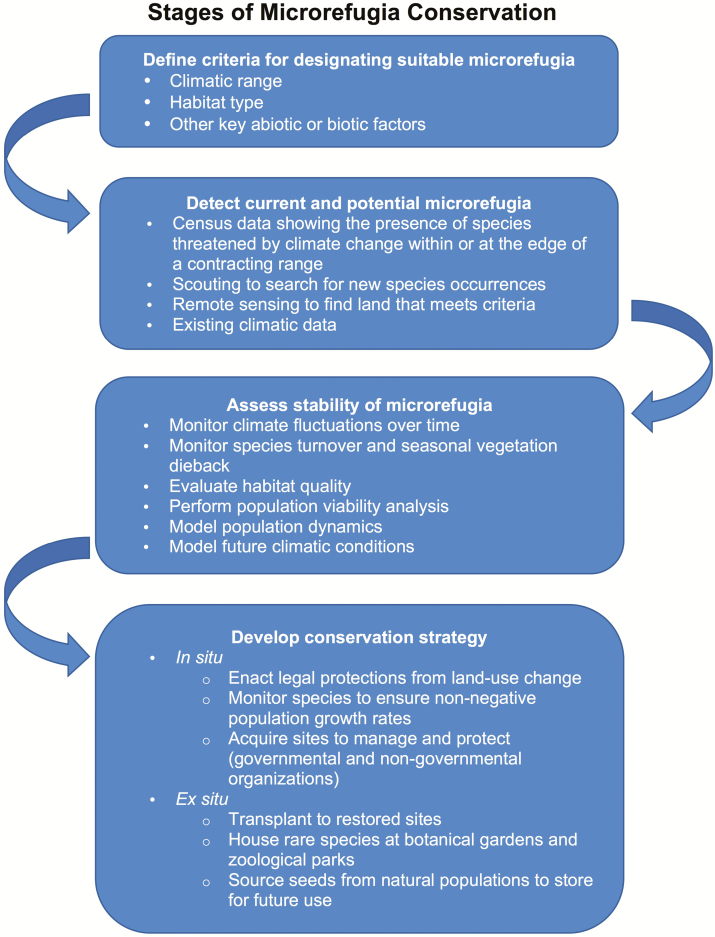
Proposed framework to identify and conserve plant microrefugia for species threatened by climate change. Microrefugia conservation is a fluid process that may not always follow these discrete steps. Multiple viable approaches exist, and steps may differ when the goal is to protect ecological communities rather than individual species.

Developing management strategies for protecting habitats can be difficult when current warming trajectories greatly differ from historical patterns (Morelli *et al.* 2016). McLaughlin and colleagues (2017) argue that identifying species-specific hydraulic refugia is not only essential for plant population persistence but also beneficial in understanding the hydraulic effects of climate change. Topographic heterogeneity creates variation in water availability across landscapes, and benign micro-environments could serve as hydraulic microrefugia for plants that require mesic habitats when the environment becomes warmer and more arid (McLaughlin *et al.* 2017). Conservationists have not yet agreed upon procedures for using microrefugia to conserve plants, nor are there many policies in place to protect microrefugia. Numerous strategies to identify and preserve microrefugia exist, but the most effective option depends on the conservation goals (Fig. 4). For example, to conserve a species threatened by drought, it may be more important to find a hydraulic refuge than a refuge with a suitable and stable climate (Fig. 4). After deciding how to define a refuge, there are multiple options for detecting stable locations that meet this criterion (Fig. 4). National-scale conservation laws may not be sufficient to protect threatened species if they do not account for the specific needs of organisms on a regional scale (Rossi *et al.* 2015).

Early efforts to preserve rare and endemic species in microrefugia hold great promise. In Valencia, Spain, researchers have established microrefuges, which are small (up to 20 hectares), actively managed areas intended to protect species and habitat types for the long-term persistence of plant populations (Laguna *et al.* 2004). Strategically located microrefuges protect more critically endangered taxa than larger natural protected areas (Laguna *et al.* 2004) while also harbouring more species than neighbouring plots of the same size without a microrefuge (Fos *et al.* 2017). Thus, managing *in situ* microrefugia is an effective conservation method.

If microrefugia are indeed havens for biodiversity, then biodiversity within microrefugia would be relatively high, even when very little land has been sampled. In a recent meta-analysis, Stein and colleagues (2014) found a significant and positive relationship between environmental heterogeneity and species richness across taxa, spatial scales and regions. Accounting for fine-resolution heterogeneity in landscapes can help identify and preserve microrefugia, especially for species imperilled by climate change. This process will become easier as researchers continue to collect demographic and trait data across diverse ecosystems to assess and predict how species, especially rare species, respond to global change.

## Conclusions

Climate change is rapidly altering ecological and evolutionary dynamics of natural communities and populations. Here, we argue that plant responses to novel suites of conditions may depend on the extent of abiotic and biotic micro-environmental variation in the landscape. Local micro-environments may already reflect climatic projections for a region if some microhabitat patches experience warmer temperatures or more or less arid conditions than average for a given site. Given that fine-grained spatial and temporal heterogeneity in environments can favour the evolution of phenotypic plasticity, pre-existing plasticity to the micro-environment may buffer local populations from decline as the climate continues to change. However, existing plasticity may not be sufficient to stabilize populations long term if future climates exceed the range of variability of historical climates. In those cases, adaptive evolution could be necessary to enable population persistence. We posit that micro-environmental variation could favour the evolution of adaptive genetic variation within local populations owing to different selective regimes over very small spatial scales. In that case, local populations might already harbour sufficient genetic variation to avoid extinction under climate change. Finally, benign local environments that reflect historical climates have sometimes facilitated survival through geological climate change, and these types of environments (e.g. natural depressions with cooler temperatures) could serve as microrefugia under anthropogenic climate change, enhancing plant conservation as climate change continues. We encourage research that explicitly incorporates microhabitat variation into studies of plant responses to climate change. Ultimately, evolution in response to historical levels of micro-environmental variation might have generated enough adaptive potential to maintain populations in the short term, allowing us more time to enact effective conservation policy.

## Supporting Information

The following additional information is available in the online version of this article—

**Table S1.** Climatic data from a high-elevation meadow location near the Rocky Mountain Biological Laboratory (latitude: 39.0315806; longitude: −107.07846, elevation: 3340 m) from the period 1 May 2016 to 31 December 2016. The Decagon 5 TM sensors recorded soil temperature and soil volumetric water content at 10 cm depth at 15-min intervals. Data are displayed in Fig. 2.

plaa005_suppl_Supplementary_SI_Table_S1
